# Consumer Attitudes and Preference Exploration towards Fresh-Cut Salads Using Best–Worst Scaling and Latent Class Analysis

**DOI:** 10.3390/foods8110568

**Published:** 2019-11-13

**Authors:** Stefano Massaglia, Valentina Maria Merlino, Danielle Borra, Aurora Bargetto, Francesco Sottile, Cristiana Peano

**Affiliations:** 1 Dipartimento di Scienze Agrarie, Forestali ed Alimentari, Università di Torino, Largo Braccini, 10095 Grugliasco, Italy; stefano.massaglia@unito.it (S.M.); danielle.borra@unito.it (D.B.); aurora.bargetto@unito.it (A.B.); cristiana.peano@unito.it (C.P.); 2 Dipartimento di Architettura, Università di Palermo, Viale delle Scienze, Edificio 14, 90128 Palermo, Italy; francesco.sottile@unipa.it

**Keywords:** best–worst scaling, consumer preferences, latent cluster analysis, fresh-cut salads, quality attributes

## Abstract

This research explored the preferences and buying habits of a sample of 620 consumers of fresh-cut, ready-to-eat salads. A best–worst scaling approach was used to measure the level of preference stated by individuals regarding 12 attributes for quality (intrinsic, extrinsic and credence) of fresh-cut salads. The experiment was carried out through direct interviews at several large-scale retail outlets in the Turin metropolitan area (north-west of Italy). Out of the total number of questioned consumers, 35% said they did not consume fresh-cut salads. On the contrary, the rest of the involved sample expressed the highest degree of preference towards the freshness/appearance attribute, followed by the expiration date and the brand. On the contrary, attributes such as price, organic certification and food safety did not emerge as discriminating factors in consumer choices. Additionally, five clusters of consumers were identified, whose preferences are related both to purchasing styles and socio-demographic variables. In conclusion, this research has highlighted the positive attitude of consumers towards quality products backed by a brand, providing ideas for companies to improve within this sector and implement strategies to answer the needs of a new segment of consumers, by determining market opportunities that aim to strengthen local brands.

## 1. Introduction

According to the United Fresh Produce Association and the Food and Drug Administration [1], fresh-cut fruit and vegetable products are defined by being minimally processed (already washed, cut, mixed and packaged) and ready for consumption [2]. These products, particularly fresh-cut salads in a bag (FCS), are a significant example of innovation and successful integration between agriculture and the food industry, focused on the satisfaction of the modern consumer and lifestyle [3,4,5]. The high variety of fresh-cut vegetables on the market allows the consumer to differentiate their choices, combining in a single product genuineness with ease of use—all traits well aligned with the modern food consumption trends of “healthy” and “time for money/time saving” [6,7,8]. The market, by not being influenced by the seasonality of the product, offers monovarietal fresh-cut products (e.g., rocket, lettuce, iceberg) and multi-varietals (mixed salads), all of them ready to eat raw or cooked (spinach, herbs, green side dishes, legume soups), of already peeled fruits (citrus, pears, apples, pineapple, etc.) and opportunely cut (in wedges, slices, cubes) produce, or complete meals sold in single-dose containers equipped with forks for eating out. The evolution of food technology has been applied both to the cultivation phase in the field, and to post-harvest management (e.g., for shelf-life prolongation) and has resulted in products offering effective services (convenience food) together with marked freshness traits, and organoleptic and sensorial characteristics unchanged with respect to the fresh product [5]. For example, the improvement of packaging technologies has allowed the development of more sustainable and practical solutions [9] enabling the consumption of these products outside the home, such as in the workplace and while traveling. Even in the Italian context the fresh-cut salads consumption is increased in accordance to consumer research of health-enhancing foods in parallel to convenience product aspect, even outside the home. In particular, at the national level and compared to their first appearance in the early 1980s, the fresh-cut fruit and vegetable sector has grown exponentially over the last decade (+376%), especially in the north of the country with a 60% share of the total [10]. As for salads in a bag, in 2017 approximately 19.4 million Italian consumers reported regularly buying these products, especially through large-scale distribution, which is reported as the main sales channel for these products. In the same year, the only Italian market for fresh cut and wrapped salads registered a +4% trend in large-scale distribution, with an increase in sales (+8.2%) for private labels especially, which hold a 60% market share [11].

The increase in consumption and the development of the fresh-cut salad market in Italy went hand in hand with the development of consumption profiles characterized by different preferences towards the different attributes that describe the product. In addition, besides responding to nutritional needs, the consumption of fruits and vegetables in general is a hedonic and gustatory experience, based on the interaction between the intrinsic/sensorial characteristics of the product (freshness, appearance, aroma). It is therefore very important that the need for “convenience” of the product is always accompanied with the maintenance of the nutritional and sensorial quality, in order to better satisfy the needs of the consumer. Among the internal and sensory attributes evaluated by the consumer in the choice and at the consumption of fresh-cut salads, we found in the literature the freshness, appearance, variety, and seasonality were of greatest importance [12,13,14]. In addition to the intrinsic aspects, the purchase act is increasingly influenced by the assessments of individuals towards the so-called attributes of belief (such as safety, nutritional benefits, origin, quality certifications, organic status, local production, and sustainability of the product) [15,16,17]. Nevertheless, a good number of consumers still consider this product too expensive, unsustainable, and of high hygienic-sanitary risk (it can be seen as a carrier of pathogens such as *Salmonella* spp., *Listeria monocitogenes*, *Escherichia coli*, and *toxoplasma)* [18,19,20,21].

Due to the heterogeneity and high variability of salads in a bag on the market, as well as the remarkable increase in the consumption of these products, the analysis of individual trends, attitudes and preferences has become very important to deciding on the marketing strategies of the operators of the sector, considering the joint evaluation of different types of attributes (extrinsic, intrinsic, credence) that characterized the considered product. In this research, it was assumed that (H1) the consumer evaluates in a differently way the attributes describing fresh-cut salads to the three above-mentioned categories. In order to verify this hypothesis, were then chosen 12 descriptive attributes of salads in bag among credence, intrinsic and extrinsic features as a result of a literature research. The declared preferences by a sample of individuals intercepted at different outlets in the Turin metro area (northwest of Italy) were analyzed by adopting the best–worst scaling (BWS) approach. The BWS, also known as maximum difference (Maxdiff), allows us to compare the degree of preference expressed towards attributes from individuals intercepted in a choice experiment through a multivariate and quantitative methodological approach. In addition, other hypotheses of our research included that could be exits a correlation between the declared preferences and the consumer socio-demographic characteristics (H2), as well as their purchasing behavior (H3). At this purpose, to supports this latter hypothesis the Latent Class Cluster analysis was made to identifying different consumer targets that were described in function of preferences, fresh-cut salads purchasing habits and socio-demographic variables.

## 2. Materials and Methods

### 2.1. Data Collection

**Figure 1:**
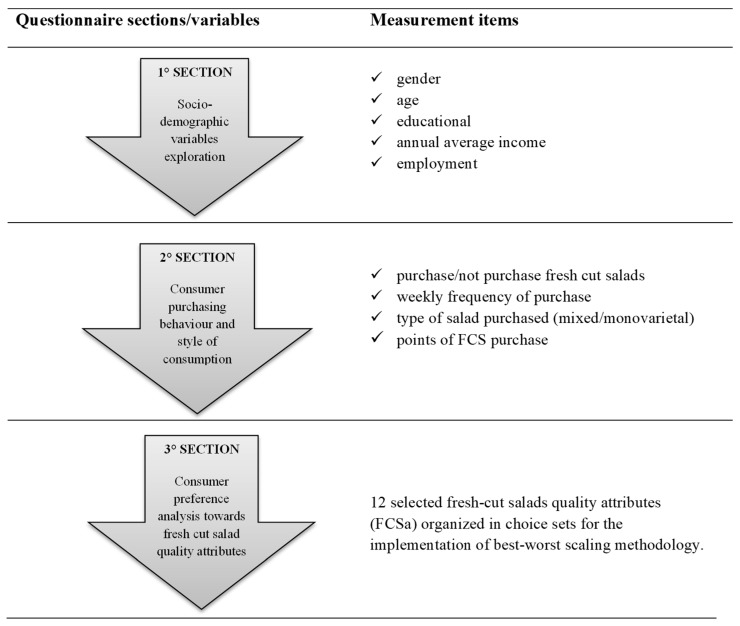
The conceptual framework of the survey.

A paper questionnaire was developed in order to explore the preferences and habits of purchase and consumption of our sample. A total of 620 individuals were selected for direct (face-to-face) interviews in different points of sale of large-scale chains (supermarkets and hypermarkets) in the metropolitan area of Turin. The survey was structured into three main sections (Figure 1) designed, respectively, to investigate the socio-demographic variables of the individuals interviewed, their habits and consumption patterns for FCS, and their declared preferences towards the 12 quality attributes (FCSa) of salad in a bag. In particular, the last section of the questionnaire was structured to implement the best–worst scaling methodology.

### 2.2. Data Analysis

The data analysis included two steps: First, we calculated the importance of each selected attribute in describing the fresh-cut salads in a bag. Secondly, the indexes of relative preference deriving from the survey on the entire sample were used for the analysis of the heterogeneity of individuals and cluster segmentation.

The BWS methodology was developed in 1992 by Finn and Louviere [22]. Compared to other methods of indirect measurement of individual preferences, the BWS allows us to overcome the limits of the range and of the ranks, which imply a high cognitive effort required by the interviewee, reducing the efficiency of the investigation [22,23]. Furthermore, even if this methodology provides an ordinary scale of qualitative attributes, it assigns a quantitative value to the individual preference level declared by the respondent (preference index).

An incomplete balanced block design (BIBD) was used. A BIBD design has the following characteristics: (1) Each attribute occurs in each comparison set at most once, and (2) each of the attributes m appears exactly *r* times through the choice sets and co-runs exactly *λ* times with other (*m*−1) attributes. The numbers *λ* and *r* are integers, and *λ* can be calculated according to the equation of *λ = r × (m* − 1*)/(k* − 1*)* [23,24,25]. The main advantage of this design is that it allows us to considerably reduce the number of comparison sets to be evaluated [24,26]. Given the set of k attributes, the BIBD has created *s* choice sets containing *m* attributes per set, which implies the constant condition *k > m*. A preferred BIBD design is symmetrical (when *s = k*), which is also known as the Youden square [23]. In the present paper, 12 attributes (*k =* 12) describing salads in envelopes (FCSa) were selected, according to different authors [27,28,29]. Both the intrinsic and extrinsic quality cues and credence attributes were selected [2,14,26] to describe the FCS (Table 1).

Using the software Sawtooth (SSI version 8.4.6, Orem, UT, USA; http://www.sawtoothsoftware.com/), the FCSa of quality (items) were implemented in the questionnaire using an experimental design BIBD characterized by the attributes of nine sets (*s*) containing four FCSa each (*m*). Furthermore, four different versions of the questionnaire were created and used for the design, and on each of them a single attribute appears three times (*r*) in different combinations. The interviewed people had to indicate, among the four attributes of each set, the characteristic they consider the most influential/important (BEST) and less influential/important (WORST) during the selection and purchase of the product. Therefore, it is assumed that the preference of an individual with respect to object A and with respect to object B is given by the frequency with which A is chosen as preferred with respect to B (Random Utility Theory) [66].

Feedbacks collected from the interviews were analyzed to obtain the raw numerical preference index for each quality attribute (average raw score, A-RS).

The methodology of BWS assumes that respondents can easily make a reliable and valid choice, since they only need to choose the options “best” and “worst” in each set of choices. By asking them repeatedly to choose the two most extreme attributes from a series of sets, we can achieve the level average of importance of each attribute with the following equation (1):

$$\mathrm{Average}\;\mathrm{Raw}\;\mathrm{Score}\;=\;\frac{\;COUNT_{best-}COUNT_{worst}}{r\times n}$$

where Count*_best_* is the total number of times each attribute has been chosen as “best “, Count*_worst_* is the total number of times each attribute has been listed as “worst”, *r* is the number of times each attribute appears, and *n* is the number of observations.

This value can be positive or negative, and the sum of all the values is equal to 0. Furthermore, the integrity analysis of the BW results was carried out according to the criteria described by Liu et al. (2018) [24].

The relative preference values per each single attribute, or the rescaled score (whose sum is equal to 100), were instead submitted to the Latent Classes Analysis (LCA) and used to identify homogeneous subgroups of the sample population in relation to the preferences of the consumers for the tested attributes [66,68,69]. This analysis was performed directly by the same software used for the best–worst analysis (Sawtooth software). In particular, the experimental design for cluster identification approached on the default division of the whole sample from 2 to 5 clusters in function of individual preferences scores. In our research, the decision to use the rescaled score for the cluster analysis was made as it facilitates the analysis and interpretation of the preferences of the single clusters, and the comparison between them [70]. The best segmentation was evaluated by analyzing the log-likelihood (LL) values and the related Bayesian Information Criterion (BIC) of each model [71,72]. In this way, the five-cluster model was selected because it is more economical and is able to provide the best segmentation corresponding to the lower LL and BIC values, as shown in Table 2.

The clusters were analyzed based on (i) the relative preference level expressed per single attribute; and (ii) the social-demographic traits and the buying habits and styles for fresh-cut salads. The standard deviation was used as a raw indicator of variability within the sample. A t-test with two queues was performed to check how the distributions in different clusters for each attribute behave. The *p*-value for each attribute was calculated for the validation of the homogeneity of the cluster segmentation. SPSS.25.0 for Windows was used for the quantitative analysis.

## 3. Results

### 3.1. Participants

A total of 35% of the intercepted subjects did not complete the questionnaire, having declared that they did not purchase FCS. Questionnaires from 400 interviewed participants were used to analyze preferences for fresh-cut salads. The respondents were mostly women (68%), represented by the 52% by individuals from 46 to 65 years old. Only 18% of the sample (*n* = 72) was single, while the rest were married (*n* = 128) with children (*n* = 200). More than half of the sample (*n* = 208) had a higher level of education, and were employees (49%, *n* = 196). Data on the sample involved in the research are reported in Table 3.

### 3.2. Analysis of Preferences

**Figure 2:**
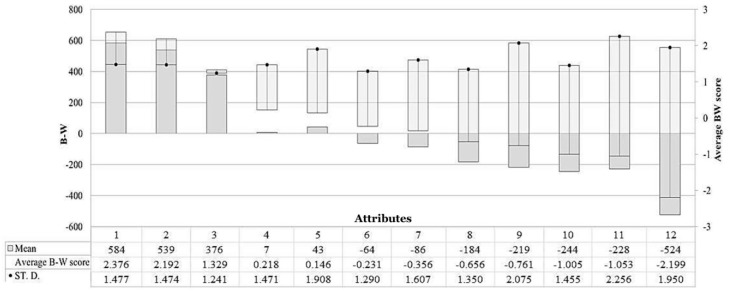
Results of the best–worst scaling analysis: for each single attribute chosen, the value of the best minus the worst (BW), the average raw score (preference level), and the standard deviation are shown. Attributes: 1—Freshness/appearance; 2—Expiry date; 3—Brand; 4—Variety; 5—Local production; 6—Labeling information; 7—Seasonality; 8—Environmental sustainability; 9—Price; 10—Organic certification; 11—Promotional offers; 12—Safety product.

The results of the BWS analysis evidence the degree of preference expressed by the interviewed participants regarding the individual attributes chosen to describe FCS. Figure 2 shows the average level of importance (A-RS) assigned to each attribute from the consumers involved in the study.

All information perceived as a favorite receives a positive score above the “0” line. In particular, the most important attributes during the purchase of the products in the analysis were, in descending order, the “freshness/appearance”, the “expiry date”, and the “brand” (Figure 2). On the contrary, the interviewed individuals attributed lower relevance to “food security” and “organic certification”, as well as “promotional offers”. The standard deviation is similar for the first two most important attributes. However, the high variability of the answers provided by respondents for the degree of importance of the factors “promotional offers”, “price” “nutritional security” and “local production” should be underlined.

In this research, the integrity of the data and their processing was tested following the methodology reported in Liu et al. (2018) [24]. According to the equation for calculating the average raw score (Equation (1)), the BW score is between −*r* × *n* (i.e., −3 × 400 = −1200) and +*r* × *n* (i.e., +3 × 400 = +1200). At the same time, the integrity of the data is provided by the equality of the sum of the number of times each attribute was chosen as the most preferred, and the sum of the number of times the same attribute was chosen as the least preferred; that is, the “times selected best”, the “times selected worst” and the values of “best–worst” for each attribute reported in Table 3. For the total sample, moreover, in the absence of missing values, both the sums are equal to *s* × *n*; that is, equal to 400 × 9 (3600). In addition, the sum of the average raw scores calculated for each attribute is equal to zero (Table 4).

.

### 3.3. Latent Class Clustering Analysis

Five groups of interviewed individuals were identified, and they were renamed according to the most relevant attributes considered for the choice of FCS. All segments differ significantly (*p-*value < 0.05) from each other with respect to five quality attributes (food safety, information on the label, mark, seasonality, and environmental impact) (Table 5). In addition, considering the socio-demographic variables, all clusters reflected the proportion between women and men already highlighted in the whole sample (on average 68% of women vs. 32% of men). However, differences between individuals belonging to different clusters emerged from the analysis of the other socio-demographic components. The first cluster, named “appearance attentional”, includes 30% of the total interviewed (120 consumers), particularly those that assigned a higher average importance to the intrinsic quality of the product (appearance and freshness) and to the expiry date. Basically, these individuals always chose the same brand of product. In particular, the rescaled score relating to the “freshness/appearance” attribute was the highest, even compared with the scores associated with the other variables for each cluster. These interviewed individuals attributed little importance to the price, to food security, and to promotional offers. From a social-demographic point of view, they were mainly worker women, usually managers of two-family households (in the case of more mature women), and of families with children (younger women). They also had an average high income, as well as educational level.

A total of 21.6% of the sample fell into the “local sensitive” group. In this case, the FCSa of experience and belief creates a union of values important for these individuals related to local products, local producers, and having a sustainable attitude from an environmental point of view; moreover, in the case of these individuals the highest RS (9.34) was related to the attribute “environmental impact”. However, the expiry date remains an important attribute for the purchase of salad in a package for these individuals, as well as seasonality, which was assessed as a discriminating variable. For this consumer, the price and promotional offers do not affect the purchase process. The social-demographic profile of this cluster is comparable to that of the “attentive appearance”, differing only in having a greater percentage of older individuals and representatives of large families. The “variety/price sensitive” (18.5%) were characterized choosing based on the intrinsic characteristics of the product (freshness, expiry date and variety), but also on price. In this case, features such as the brand, local origin, environmental impact, and food safety appeared not very relevant attributes. This segment had 34% of individuals under 35 years old, also in this case consisting mainly of women managing the purchasing aspects in young couples, or families with one child. They are often employees, with a high level of education but with an average low income.

A total of 16.3% of the sample was represented by “health safeguard” consumers, for whom a marked importance for the attribute “food safety” emerged, with the highest RS value among the different clusters (RS = 13.6). In this case, the credence attribute is positively related to the freshness of the product, but also to the brand, which is considered synonymous with guarantee. Even in this case, the expiry date is one of the discriminating factors for the choice. These individuals are distinguished by a majority of mature women (20% were over 65 years old), representing the absolute oldest cluster. They had an average level of education, 27% were retired, and they had a low-medium income. The “value for money” group place the quality/price ratio in the foreground as they select a product, with an approach to maximizing the convenience of the product, choosing quality and service. These individuals represented 13.6% of the sample and were characterized by 41% being under 35 years old, thus representing the youngest cluster. They were workers, with an average level of education and a low income on average.

**Figure 3:**
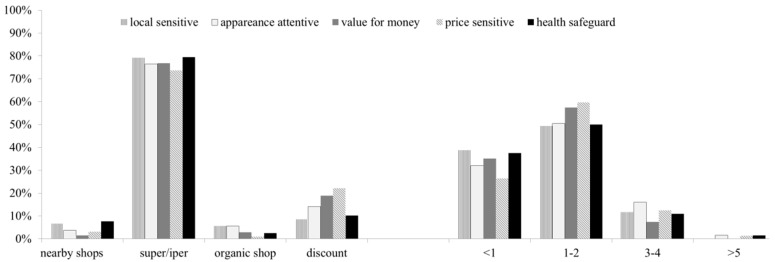
Places and weekly frequency of purchase of the FCSa, as declared by individuals belonging to the different clusters.

The sites where the consumers belonging to the five clusters typically purchase showed no significant differences: a general dominance of super and hypermarkets were utilized for FCSa in all five clusters (Figure 3). Discount stores represented the second most selected choice by the consumers, with a slight dominance for the “price sensitive” and “value for money” groups. Moreover, among the consumers who chose to buy packed salads at the nearby shops, the individuals in the “local sensitive” and “health safeguard” dominated. As for the weekly purchase frequency of FCS, more than half of the individuals in each cluster bought them 1–2 times a week, while an average of 30% bought them just less than once a week.

## 4. Discussion and Conclusions

The FCS sector in Europe, over the last 10 years, has shown and maintains exponential growth in sales and consumption. In the Italian context, and especially in the northern regions, FCS has now become a product ordinarily purchased, with a constant presence in the family cart [10,73].

In this exploratory research, it appears worth pointing out that 36% of the consumers (620) claimed not to buy FCS. Therefore, despite the increasing trend of the market, a good number of consumers still exclude this item from their purchase choices, probably for reasons deriving from issues related to the economic and/or environmental unsustainability of the product [33,39,74] or due to health and hygiene safety concerns [2,60].

However, a good number of consumers expressed appreciation for the product, emphasizing the importance of qualitative attributes linked to the freshness and appearance of the salads [5] and to the service provided. The BW approach allowed to confirms the our first hypothesis (H1) identifying and quantifying the individuals preferences regarding the attributes of FCS, underlining how the intrinsic quality of the product, probably assessed by looking at the expiration date, as well as brand experience and loyalty, especially affect consumer purchases.

Although, in general, little attention appeared to be paid to the information on the label, as already highlighted by several authors [13,74,75,76], the consumer tends to give particular importance to the expiration date, an attribute emerging as discriminant both in the total sample and in the different clusters. According to the research by Van Boxstael et al. (2014) [77], the expiration date has been associated with the healthiness and shelf-life of the product in a refrigerator at home, and indirectly with the risk of a decrease in quality.

Unlike the research carried out by Stranieri and Baldi [5] in Lombardy on other products of animal origin [28,76], in this case the organic certification, the seasonality, and local production play a less important or totally irrelevant role for consumer choice.

The cluster analysis has, however, identified five consumer clusters each one defined by different preferences level towards the 12 selected quality attributes and different socio-demographic variables, confirming our research hypothesis (H2). On the contrary, our results were in contrast to the starting hypothesis (H3) excluding a correlation between cluster declared preferences and the individuals purchasing habits in terms of the choice of fresh cut salads sales channels and it weekly frequency of purchase.

First a group of consumers were identified (21.6%) who are focused on such aspects as local products, along with the brand and the expiration date, as well as environmental sustainability and seasonality, in product selection. The choice of local products and, therefore, of a short food chain is connected to the selection of a trademark, or tied to local producers/distributors, enhancing their products. We note the importance of social aspects and economic sustainability for these individuals, with a more conscious awareness of environmental impacts, mainly related to the water footprint [73] resulting from the FCS production process.

The larger cluster (30%) was that of “appearance attentive” consumers, reflecting the preferences expressed by the sample as a whole. For these individuals, the intrinsic quality of the product was fundamental for the purchase and was connected to a specific brand, which was also a guarantee of safety. The low price, as a result of a promotional offer, was probably negatively interpreted by the consumer who is “appearance attentive” as being synonymous with a poor quality product, probably close to the expiration date [68,78].

The “health safeguard” (16.3%) group are consumers for whom a marked importance emerges for the attribute “food safety”, which is positively correlated with the freshness of the product, but also to the brand, which is synonymous with guarantee. For this cluster, the socio-demographic component that mostly distinguishes it from the other clusters has a great impact [42,79]; in fact, these subjects had a greater percentage of aged women (20% were over 65 years old), representing the oldest cluster. This result demonstrates how mature women seek healthy and safe food for their own health and not only for their family; these individuals being more likely to be single or represented by two-component nuclei.

The least representative cluster of the total sample was that of “value for money”; those who were attentive to the quality/price ratio during the choice of the product, with a view to maximizing convenience, for a product that on average has a higher price rather than fresh, choosing not to give up quality and service. From a socio-demographic point of view, our analysis shows that gender is not a discriminating feature in salad choices; however, age reflects the orientation of the choices, especially in terms of health safeguards (individuals over 65 years old) and in clusters where the price and the search for promotional offers affect the choice (young clusters). These trends affirm the orientations and attitudes of young people (price sensitive) and older individuals during decisions to purchase other products such as milk [72] or meat [45]. Even income, as stated by Andreyeva et al. [80] and Webber et al. [81], affects choices, and above all the greater tendency to search for offers and products on promotion, which is also the case for FCS. The results of this research confirm the supremacy of large retailers as a place of purchase, and the tendency of price and promotional offer sensitive individuals to search for the most convenient product at discount stores. In addition, although these are small percentages of individuals, the tendency for the “local sensitive” and “health safeguard” clusters to buy range-based salads at nearby shops emerges. The latter result underlines the importance of local brands that are emerging in small shops. In Piedmont, for instance, it is possible to find several private brands providing a local, safe product, linked to the regional context, in small shops of distribution in big cities. This positive response, even if from a small percentage of consumers, could be accepted by producers for the diffusion of proven brands, and not only in large-scale retail trade.

In conclusion, this research allowed us to identify the criteria for selection used by consumers in Piedmont during the purchase of fresh-cut salads. This research, in fact, allows to provide an important contribution to the current literature, as it has allowed to correlate the socio-demographical characteristics to the individual declared preferences (through a quantitative methodology) about a product little explored, such as salads in bags. However, even if the analyzed geographical context has allowed us to gather a representative number of answers, since the metropolitan city of Turin is one of the areas in which the consumption of fresh-cut salads is widespread, the limited range represents a critical limit which could also be extended to other regions of central and southern Italy. Further research would make it possible to overcome a further limit of this work given by the lack of homogeneity of the sample (especially women, unbalanced mainly towards an age group). Moreover, given a good percentage of people are still skeptical and reluctant to purchase this product, it would be interesting to investigate the reasons behind these consumers choosing a fresh product instead, evaluating their consumption patterns and also extending the investigative context to a non-metropolitan area (rural context).

In conclusion, this research provides an important contribution to the literature because it gives suggestions for improvement to companies in order to develop new marketing strategies. For an area like this, which is strongly related to the trust consumer-brand, it could be beneficial to deeply analyze the relationship between producers and private labels, which currently absorbs the majority of the fresh-cut vegetable market. In this regard, a starting point could be encouraging investments into the communication campaigns of private brands, which are currently absent or minimized to a few distributors who also concentrate their advertising messages during the summer or just for the launch of new products.

## Figures and Tables

**Table 1:** The 12 fresh-cut salad attributes (FCSa) selected for the best-worst scaling (BWS) methodology implementation. Each item is classified into the intrinsic, extrinsic and credence attribute categories and some references are reported for single characteristics.

Attributes category	Fresh-cut salads attributes (FCSa)	References
**Credence attributes**	Organic certification	[30,31,32]
	Local production	[33,34,35,36,37]
	Environmental sustainability	[9,38,39,40,41]
	Safety product	[42,43,44,45]
**Intrinsic quality cues**	Seasonality	[41,46,47]
	Variety	[36,47,48]
	Freshness/appearance	[5,49,50,51]
**Extrinsic quality cues**	Labeling information	[52,53]
	Brand	[54,55,56]
	Price	[27,28,57,58,59,60,61,62,63,64,65,66,67,68,69]
	Expiration date	[60,61,62,63]
	Promotional offers	[64,65]

**Table 2:** Models (segmentations) of the latent class analysis adapted to the individual preference indices expressed for the 12 attributes of salads in envelopes.

Template	LL	BIC	Chi-Square	Relative Chi-Square
Two-cluster model	−8074.19	16,352.67	3814.25	165.83
Three-cluster model	−7920.02	16,150.90	4122.60	117.79
Four-cluster model	−7808.59	16,034.63	4345.45	92.46
Five-cluster model ^1^	−7718.68	15,961.39	4525.28	76.70

Note: LL = Log-likelihood; BIC = Beyesian Information Criterion. ^1^ This model was chosen as corresponding to the lowest value of BIC and chi-square (relative).

**Table 3:** Socio-demographic traits of the sample (*n* = 400).

Features	Categories	Percentages
Sex	Women	68%
	Men	32%
Age (years-old)	18–25	6%
	26–35	13%
	36–45	14%
	46–55	27%
	56–65	25%
	over 65	15%
Education	Elementary school degree	2%
	Middle school degree	17%
	High school degree	51%
	University degree	30%
Employment	Student	4%
	Employee	49%
	Independent worker	13%
	Retired	22%
	In search of work	7%
	Housewife	5%
Annual average income	<25,000 euros	35%
	25,000–40,000 euros	43%
	40,000–60,000 euros	17%
	>60,000 euros	5%
Number of family members	1	18%
	2	32%
	3	24%
	4	20%
	>4	6%

**Table 4:** Test results on the integrity of the data based on the sum of the values of “times selected best”, “times selected worst”, and “BW” (best-minus-worst).

Label	Times Selected Best	Times Selected Worst	B-W
Freshness/appearance	653	69	584
Expiry date	619	80	539
Brand	463	87	376
Variety	285	278	7
Local production	334	291	43
Labeling information	223	287	−64
seasonality	240	326	−86
Environmental sustainability	117	301	−184
Price	182	401	−219
Organic certification	137	381	−244	
Promotional offers	222	450	−228
Safety product	125	649	−524
Sum ^1^	3600 (a)	3600 (b)	0 (c)

^1^ The integrity of the results is demonstrated if (*a* = *b* = *s* × *n*); if ∑(B−W)=0.

**Table 5:** Latent class clustering analysis: For each cluster; the name, size (% of the sample) and *p-*values are indicated. The individual clusters are also described according to the socio-demographic characteristics of the individuals.

Name of Cluster	Attention Appearances	Local Sensitive	Variety/Price Sensitive	Health Safeguard	Value for Money	*P*-Value ^1^
Size Group	30.0%	21.6%	18.5%	16.3%	13.6%	
Attributes	Rescaled Score (Standardized Degree of Preference) ^1^					
Food security	1.07	0.98	2.10	13.67	1.60	ns .
Information on the label	5.90	9.44	3.06	6.76	5.25	**
Manufacturer/retailer brand	15.80	16.34	5.78	12.49	11.13	*
Expiry date	19.22	12.66	18.50	12.30	15.26	***
Organic certification	3.71	8.02	1.89	5.17	2.45	*
Local origin	6.92	16.29	1.91	8.97	5.85	*
Price	2.49	1.61	15.28	3.72	13.85	ns .
Appearance/freshness	22.76	9.57	20.11	14.57	13.21	**
Seasonality	7.12	10.60	2.20	3.92	4.99	*
Promotional offers	1.72	1.17	12.77	3.44	18.07	ns .
Environmental impact/attention to the environment	4.76	9.34	1.35	5.46	5.44	*
Type/variety	8.52	3.97	15.06	9.54	2.90	*
	Social-Demographic Variables					
Gender						
Woman	68%	65%	67%	74%	63%	**
Man	32%	35%	33%	26%	37%	**
*Age*						
18–25	4%	6%	7%	6%	11%	**
26–35	11%	11%	22%	3%	17%	**
36–45	10%	9%	25%	16%	13%	**
46–55	29%	26%	29%	30%	20%	***
56–65	30%	28%	11%	25%	28%	**
over 65	15%	20%	6%	20%	11%	**
Family size (n. Components)						
1	22%	20%	18%	13%	11%	***
2	31%	29%	36%	31%	33%	***
3	20%	28%	25%	25%	26%	***
4	21%	19%	15%	20%	24%	**
5	6%	2%	6%	9%	6%	*
6	0%	1%	0%	2%	0%	ns .
Education						
Elementary high school	2%	2%	0%	0%	2%	ns .
Lower average license	16%	14%	7%	30%	22%	*
Upper middle school license	51%	54%	56%	53%	37%	***
Graduation	31%	29%	38%	17%	39%	**
Occupation						
housewife	5%	6%	4%	5%	6%	***
employee	49%	43%	61%	47%	47%	***
in search of work	5%	6%	10%	8%	11%	**
Self-employed	15%	13%	13%	11%	9%	**
retired	25%	29%	6%	27%	19%	***
student	2%	2%	6%	3%	8%	*
*Income (000 Euro)*						
<25	31%	32%	42%	34%	39%	***
25–40	40%	52%	42%	44%	39%	***
40–60	22%	15%	13%	16%	17%	***
> 60	7%	1%	4%	6%	6%	**

Note: *** *p-*value < 0.001; ** *p-*value < 0.01; * *p-*value < 0.05; ns .: *p-*value > 0.05. ^1^ The values in italics of the rescaled scores (degree of importance) correspond to the most important attributes for each cluster regarding choosing fresh-cut salads in a bag (FCS).
